# Effects of lysed *Enterococcus faecalis* FK-23 on experimental allergic rhinitis in a murine model

**DOI:** 10.7555/JBR.26.20120023

**Published:** 2012-05-09

**Authors:** Luping Zhu, Takashi Shimada, Ruoxi Chen, Meiping Lu, Qingzhao Zhang, Wenmin Lu, Min Yin, Tadao Enomoto, Lei Cheng

**Affiliations:** a Department of Otorhinolaryngology, the First Affiliated Hospital, Nanjing Medical University, Nanjing, Jiangsu 210029, China;; b International Centre for Allergy Research, Nanjing Medical University, Nanjing, Jiangsu 210029, China;; c Central Research Laboratories, Nichinichi Pharmaceutical Corporation Ltd, Mie 518-1417, Japan;; d NPO Japan Health Promotion Supporting Network, Wakayama 640-8558, Japan.

**Keywords:** allergic rhinitis, probiotics, *Enterococcus faecalis*, cytokines, eosinophils, regulatory T-lymphocytes, mice

## Abstract

In the current study, we sought to investigate whether lysed *Enterococcus faecalis* FK-23 (LFK), a heat-killed probiotic preparation, attenuated eosinophil influx into the upper airway and had immunomodulatory activity in a murine allergic rhinitis model. Eighteen BALB/c mice were divided into three groups; the ovalbumin (OVA)-sensitized/challenged group, which received saline orally for 6 weeks (OVA group), the OVA-sensitized/challenged group, which received LFK orally for 6 weeks (LFK-fed group), and the non-sensitized group, which received saline for 6 weeks (saline control group). Nasal rubbing and sneezing were monitored during the study. After the final challenge, interleukin (IL)-4, interferon (IFN)-γ, and OVA-specific IgE levels in the sera and splenocyte culture supernatants were determined, eosinophilic infiltrate into the upper airway was quantified, and splenic CD4+CD25+ regulatory T cells (Tregs) were examined by flow cytometry. We found that nasal rubbing was significantly reduced in LFK-fed mice compared to the OVA group on d 27 and 35, and sneezing was significantly inhibited by LFK administration for 35 d. LFK-fed mice had significantly less eosinophil influx into the nasal mucosa than the OVA group. There were no significant differences between the LFK-fed group and OVA group in the serum and splenocyte culture supernatant levels of IL-4, IFN-γ, and OVA-specific IgE. Interestingly, the LFK-fed mice had a significantly greater percentage of splenic CD4+CD25+ Tregs than OVA group. Our results indicate that oral administration of LFK may alleviate nasal symptoms, reduce nasal eosinophilia, and increase the percentage of CD4+CD25+ Tregs in experimental allergic rhinitis.

## INTRODUCTION

The utilization of probiotics to promote human health has been proposed for many years[1]. Several studies have demonstrated that various strains of probiotics, particularly lactic acid bacteria, are beneficial in the treatment of intestinal inflammatory conditions[2],[3]. In addition to the effects in the gut, there is increasing evidence that probiotics may be beneficial in the regulation of systemic immune responses[4],[5] and in the resistance to the development of allergies[6],[7]. This has led to increasing interest in the role of probiotics in the protection against the manifestations of allergic disease. Clinical trials have demonstrated that certain lactic acid bacteria strains have a role in the treatment and prevention of early atopic dermatitis in children[8]-[10] and that other strains could reduce nasal symptoms and improve the quality of life in patients with allergic rhinitis[11],[12]. Animal experiments have demonstrated that certain lactic acid bacteria strains, e.g., *Lactobacillus reuteri* (*L. reuteri*) and *L. casei*, can attenuate allergic airway responses[13],[14] and protect against respiratory pathogens[15].

A few studies have demonstrated that the effects of probiotics on allergy are strictly strain dependent and that strains of the same species might have opposite effects[16]. Hence, the choice of strain and the timing of the application are crucial for tolerance induction. There is still much to learn about the diverse immune responses elicited by different bacterial strains. We focused our interest on one strain of lactic acid bacteria, *Enterococcus faecalis* FK-23. A previous study demonstrated that heat-treated FK-23 has immunoenhancing effects that include increasing cell-mediated immunity, humoral immunity, monocyte/macrophage function, and natural killer cell activity in non-sensitized mice[17]. A heat-killed and lysed preparation of FK-23 (LFK) effectively inhibits allergen-induced peritoneal eosinophil accumulation[18] and appears to have the ability to enhance immune modulation in allergen-sensitized mice[19]. LFK administration may also improve the intestinal ecosystem after it has been disturbed by antibiotic treatment and may prevent the subsequent development of atopy[19]. Therefore, it was inferred that LFK may be effective against the allergic diseases in which eosinophils are involved. To further investigate the ability of LFK to attenuate allergen-induced eosinophil influx into the upper airway and the underlying immunomodulatory mechanisms, an ovalbumin (OVA)-sensitized murine model of allergic rhinitis was used in this study.

## MATERIALS AND METHODS

### Experimental animals

Four-week-old female BALB/c mice were purchased from the Laboratory Animal Center of Nanjing Medical University. The experimental mice were housed in an air-conditioned animal room at a temperature of 22±2°C and a humidity of 60%±5%, under specific pathogen-free conditions, and with a 12-h light-dark cycle. The mice were fed a standard diet and tap water that had been filtered through a pure water filter. The study protocol was approved by the local institutional review boards at each author's affiliated institutions. Experiments were undertaken in accordance with the regulations for the care and use of experimental animals of the Chinese Association for Laboratory Animal Sciences.

### Probiotic preparations

An LFK powder preparation was provided by the Nichinichi Pharmaceutical Co., Ltd. (Mie, Japan) and was prepared as described previously[18],[19]. The LFK preparation was suspended in physiological saline before it was administered to mice orally.

### Murine allergic rhinitis model

**Fig. 1:**
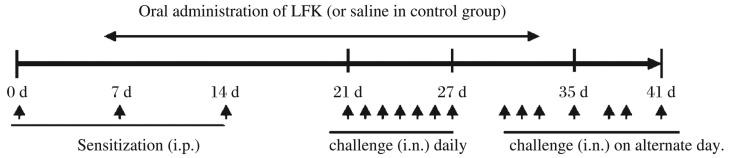
Sensitization and challenge protocols for the experimental murine model. BALB/c mice were divided into three groups: ovalbumin (OVA)-sensitized/challenged and orally administered saline (OVA group), OVA-sensitized/challenged and orally administered LFK (LFK-fed group), and non-sensitized and orally administered saline (saline control group). Mice in the OVA group and the LFK group were sensitized by the intraperitoneal (i.p.) injection of 0.1 mg OVA and 2 mg Al(OH)_3_ in a volume of 0.2 mL on d 0, 7, and 14 and were subsequently challenged intranasally (i.n.) daily with 20 µL OVA solution (50 mg/mL) on d 21 to 27 and on alternate days from d 29 to 41. The LFK-fed mice were fed 60 mg LFK, and the mice in the control group were orally administered 0.5 mL saline during the period of sensitization and challenge.

BALB/c mice were divided randomly into three groups: 1) the OVA-sensitized/challenged and orally administered saline (OVA group); 2) the OVA-sensitized/challenged and orally administered LFK (LFK-fed group); 3) the non-sensitized and orally administered saline (saline control group). Each group contained six mice. The procedures for OVA sensitization and challenge are summarized in ***Fig. 1***. Briefly, 0.2 mL normal saline containing 0.1 mg OVA (Sigma, St. Louis, MO, USA) and 2 mg aluminum hydroxide (Sigma) was intraperitoneally injected into mice on d 0, 7, and 14. The intranasal challenge was performed by nasal instillation of 20 µL OVA solution (50 mg/mL) daily from d 21 to 27, and subsequent intranasal challenge was performed on alternate days from d 29 to 41. Meanwhile, LFK (60 mg/0.5 mL/mouse) was administered orally every day to the experimental mice during the sensitization period of 6 weeks. In contrast, the OVA and saline control groups were subjected to daily oral administration of 0.5 mL saline during this 6-week period.

### Evaluation of nasal symptoms

Nasal symptoms that included nasal rubbing and sneezing were monitored on d 21, 27, and 35 after nasal provocation with OVA. The mice were immediately placed into an observation cage (one animal per cage) after intranasal challenge, and nasal rubbing and sneezing behaviors were counted for 10 min.

### Nasal histology analysis

Nasal mucosa samples from the turbinate tissue of mice (*n* = 4 for each group) were anatomically separated on d 41, fixed with 10% formalin for 24 h, decalcified in EDTA for 1 week, dehydrated, and embedded in paraffin. Fixed and embedded tissues were cut into 5-µm-thick coronal sections for hematoxylin-eosin (H&E) staining. The histological assessment was performed by light microscopy (magnification, ×200). Eosinophils in the nasal mucosa were counted at ×200 magnification.

### Measurement of cytokines and OVA-specific IgE

Blood samples were collected from the mice on d 41 (*n* = 5 for each group). After centrifugation, the serum was separated and stored at -20°C for interleukin (IL)-4 and interferon (IFN)-γ measurement. Spleens were removed, and single-cell suspensions were prepared by gently pressing the spleen through a sterile 70-micron nylon cell screener under aseptic conditions on d 41. Splenocytes were suspended in RPMI 1640 (Invitrogen, Carlsbad, CA, USA) medium supplemented with 10% heat-inactivated fetal calf serum (PAA, Australia), 100 U/mL penicillin, and 100 mg/mL streptomycin (Invitrogen), and 1×106 cells/mL were seeded in 48-well flat-bottom plates for each group. Cells were incubated in RPMI 1640 complete medium with OVA allergen (0.1 mg/mL) at 37°C for 48 hours in a 5% CO2 incubator. Supernatants were harvested and stored at -20°C for cytokine measurement.

The serum and splenocyte culture supernatant levels of IL-4, IFN-γ (R&D Systems Inc., Minneapolis, MN, USA), and OVA-specific IgE were detected in duplicate by commercial ELISA kits (Shibayagi,Co., Ltd., Gunma, Japan) in accordance with the manufacturer's recommendations. The limit of detection was 4 pg/mL for IL-4, 7 pg/mL for IFN-γ, and 1.88 U/mL (2.44 ng/mL) for OVA-specific IgE assay.

### Flow cytometric analysis

For the analysis of CD4+CD25+ regulatory T cells (Tregs), a single-cell suspension obtained from a spleen was resuspended at 1×106 cells/mL and incubated with a mixture of anti-CD3, anti-CD4, and anti-CD25 antibodies (eBioscience, San Diego, CA, USA) for 30 min in the dark. Cells were then washed twice with PBS, fixed, and analyzed with a FACScan flow cytometer (BD Immunocytometry, Franklin Lakes, NJ, USA). CellQuest software was used for data acquisition and analysis.

### Statistical analysis

SPSS 17.0 software (SPSS Inc, Chicago, IL, USA) was used for statistical analysis. All values were expressed as mean±SEM. A one-way analysis of variance was used to calculate the differences between the three groups. An unpaired Student's *t*-test (two-tailed) was performed between two groups when equal variance was assumed. In situations where equal variance was not assumed, a nonparametric test (Mann-Whitney U-test or Kruskal-Wallis test) was used. *P* values of less than 0.05 were considered statistically significant.

## RESULTS

### LFK alleviates nasal rubbing and sneezing in LFK-fed mice with allergic rhinitis

**Fig. 2:**
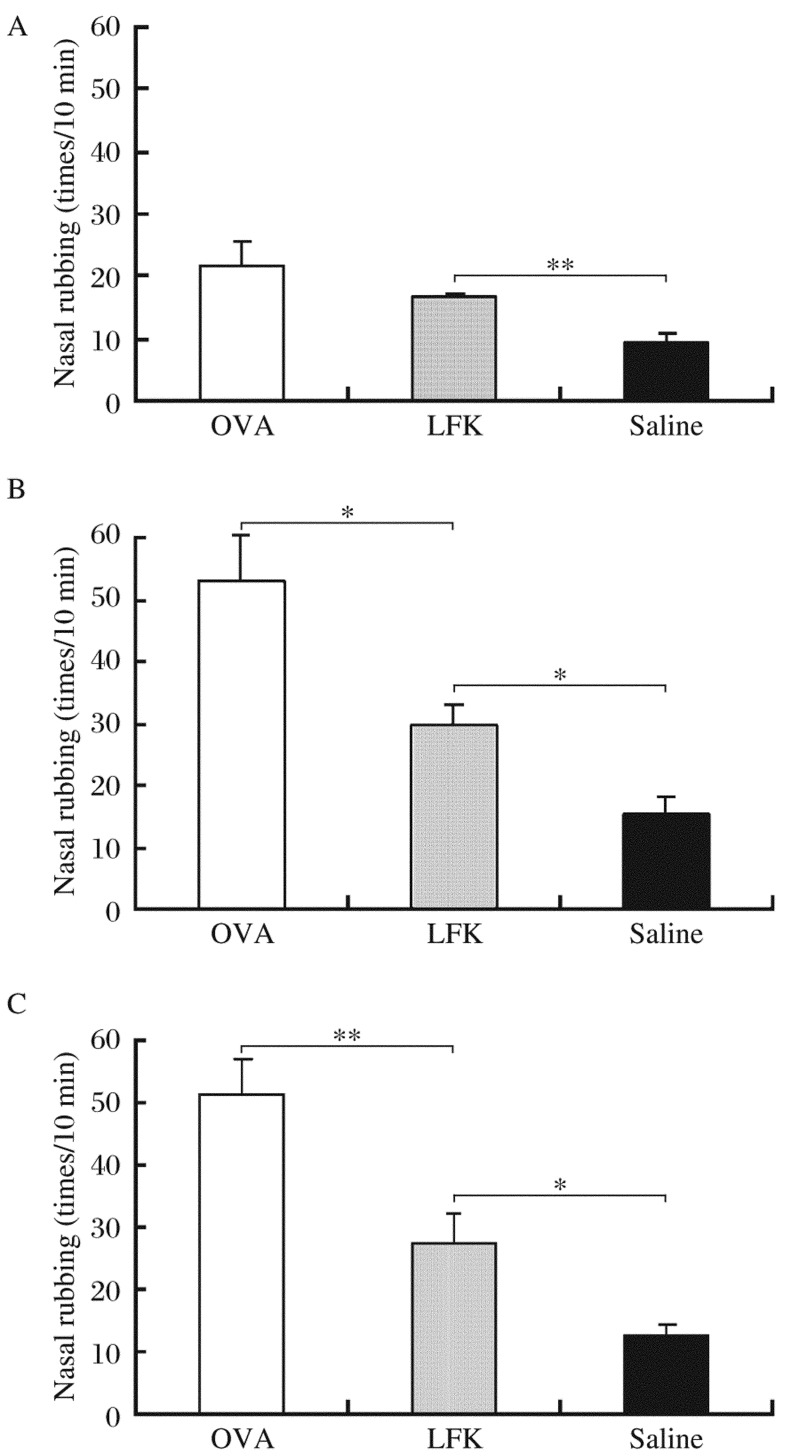
Effects of 35 consecutive days of LFK administration on nasal rubbing. Episodes of nasal rubbing was counted for 10 min after nasal ovalbumin (OVA) challenge on d 21 (A), d 27 (B), and d 35 (C). Each column and vertical bar shows the mean±SEM (n=5 each group). *P < 0.05; **P < 0.01.

Nasal rubbing increased gradually after intranasal challenge in the OVA group and was significantly alleviated in the LFK-fed mice compared to the OVA group on d 27 (*P* = 0.015) and d 35 (*P* = 0.008) but not on d 21 (*P* = 0.289) (***Fig. 2***). When compared to the saline control group, nasal rubbing of the LFK-fed mice was significantly different on d 21 (*P* = 0.005), d 27 (*P* = 0.014), and d 35 (*P* = 0.041).

**Fig. 3:**
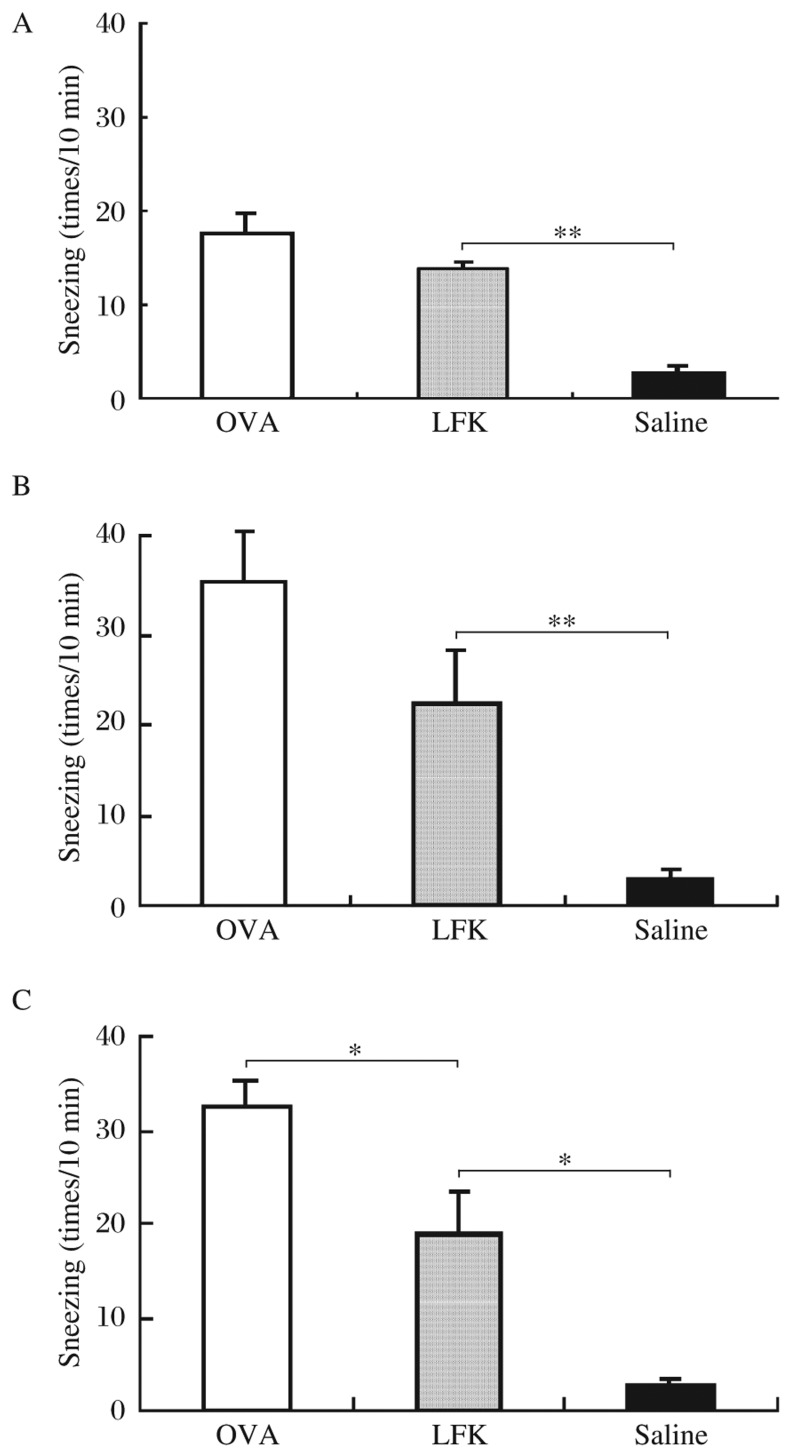
Effects of 35 consecutive days of LFK administration on sneezing. Nasal sneezes were counted for 10 min after nasal ovalbumin (OVA) challenge on d 21 (A), d 27 (B), and d 35 (C). Each column and vertical bar shows the mean±SEM (*n* = 5 each group). *P < 0.05; **P < 0.01.

The oral administration of LFK caused no inhibition of sneezing on d 21 (*P* = 0.304) and d 27 (*P* = 0.103) as there was no difference in sneezing between the LFK-fed group and the OVA group at those time points (***Fig. 3***). However, the administration of LFK for 35 consecutive days (i.e., on d 35) did significantly inhibit sneezing (*P* = 0.045). Compared to the sneezing of the saline control group, the sneezing of the LFK-fed mice was significantly different on d 21 (*P* < 0.001), d 27 (*P* = 0.002), and d 35 (*P* = 0.026).

### LFK attenuates eosinophil infiltration in the nasal mucosa of LFK-fed mice with allergic rhinitis

**Fig. 4:**
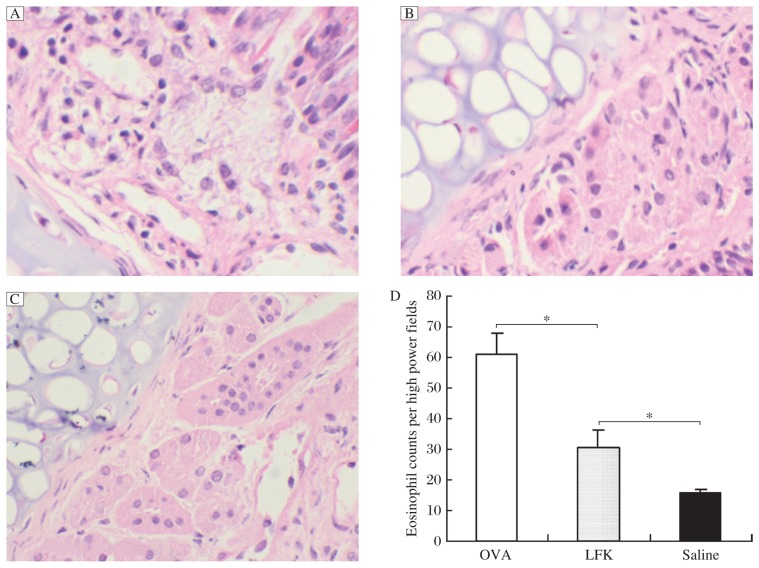
Representative H&E stained sections of the nasal turbinate tissue of the ovalbumin (OVA) group (A), LFK-fed group (B), and saline control group (C) (magnification, ×200). Nasal mucosa samples were anatomically separated on d 41, fixed with 10% formalin for 24 h, decalcified in EDTA for 1 week, dehydrated, and embedded in paraffin. Increased eosinophilic infiltration in the nasal submucosa and lamina propria was apparent in the nasal turbinate tissue of the OVA control mice compared to that of the saline control mice, whereas the eosinophilic cellular infiltrate was decreased in the LFK-fed group compared to the OVA group. D: The statistical analysis of the number of eosinophils in three groups (*n* = 4). Each column and vertical bar shows mean±SEM. *P < 0.05.

H&E staining of sections of nasal mucosa indicated an increased influx of eosinophils after the final challenge in the OVA group compared to that in the saline control group (*P* = 0.001) (***Fig. 4***), which confirmed that the nasal challenge with OVA was effective. Although the LFK-fed mice had a significantly reduced eosinophil count in the nasal mucosa compared to the OVA group (*P* = 0.014), the LFK-fed mice still had significantly more eosinophils than the saline control group (*P* = 0.044).

### Effects of LFK on serum levels of cytokines and OVA-specific IgE

After the final challenge with OVA, the serum levels of IL-4 (*P* = 0.008) and OVA-specific IgE (*P* < 0.001) were significantly increased in the OVA group compared to the saline control group (***Table 1***). The serum IFN-γ titer in the OVA group was below the limit of detection. Serum IFN-γ was detectable in the mice that were supplemented with LFK for 6 weeks, but these mice still had a serum IFN-γ titer that was significantly decreased compared to the saline control mice (*P* = 0.013). IL-4 was decreased, albeit not significantly, in the LFK-fed mice compared to the OVA group (*P* = 0.207). Likewise, LFK-fed mice had a reduction in the serum OVA-specific IgE titers compared to the OVA group, but this difference was not significant (*P* = 0.086). There were no significant differences between the LFK-fed group and OVA groups in the splenocyte culture supernatant levels of IL-4 and IFN-γ (both *P* > 0.05). The OVA-specific IgE titer was below the limit of detection in all groups (***Table 1***).

**Table 1:** The concentrations of IL-4, IFN-γ, and ovalbumin (OVA)-specific IgE in the serum and culture supernatants determined by ELISA.

Group	*n*	IL-4 (pg/mL)	IFN-y (pg/mL)	OVA-sIgE (ng/mL)
Serum				
OVA	5	58.68 ± 6.59**	n.d.	17.72 ±1.86
LFK-fed	5	45.69 ± 1.42**	14.83 ±0.99*	11.28 ± 2.05^#^
Saline control	5	20.22 ±1.75	37.12±1.65	n.d.
Supernatant				
OVA	4	24.26 ± 0.17	11.21 ±1.00*	n.d.
LFK-fed	4	18.34 ± 3.35	15.05 ± 1.76*	n.d.
Saline control	4	n.d.	67.63 ± 11.67	n.d.

*P < 0.05, **P < 0.01, as compared to the saline control. ^#^*P* = 0.086, as compared to the OVA control, n.d.: not detected.

(mean±SEM)

### Effects of LFK on CD4^+^CD25^+^ Tregs in the spleen

**Fig. 5:**
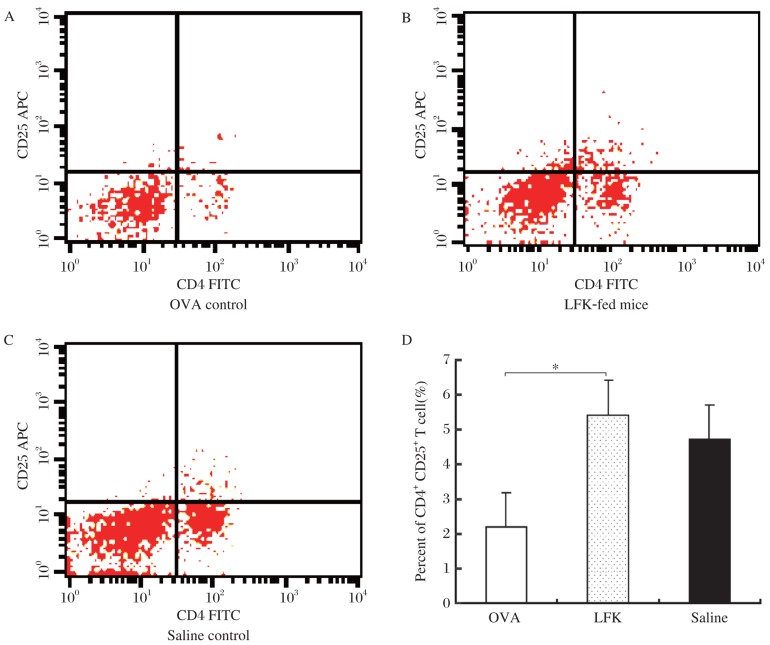
Effects of orally administered LFK on the percentage of splenic CD4^+^CD25^+^ Tregs. BALB/c mice were injected intraperitoneally on d 0, 7, and 14 with 0.1 mg ovalbumin (OVA) and 2 mg Al(OH)_3_ in a total volume of 0.2 mL. The mice were orally administered LFK (60 mg/0.5 mL) daily for 41 d. Spleens were removed within 24 h after the final nasal challenge on d 41, splenocyte single-cell suspensions were prepared, and the percentages of splenic CD4^+^CD25^+^ Tregs in OVA group (A), LFK-fed group (B), and saline control group (C) were measured by flow cytometry. D: The data shown are representative of three independent experiments. Each column and vertical bar shows the mean±SEM (*n* = 3). *P < 0.05.

The oral administration of LFK for 6 weeks led to a significant increase in the percentage of CD4+CD25+ Tregs compared to the OVA group (*P* = 0.046, ***Fig. 5***).

## DISCUSSION

In the present study, we used an OVA-sensitized mouse model of allergic rhinitis to determine that daily oral administration of LFK, a probiotic preparation of heat-killed and lysed *Enterococcus faecalis* FK-23, for 5 weeks was able to alleviate nasal allergic symptoms at the time of sensitization and challenge. Firstly, to test whether the sensitization is successful, we detected the serum total IgE by semiquantitative analysis by ELISA kit (data not shown), the result showed that total IgE was strongly positive for the OVA mice, and it was negative for the saline control mice. That confirmed that the allergy model was created.

Therefore, we analyzed the nasal turbinate tissue of mice and found that compared to the OVA group, LFK-fed mice showed decreased eosinophilic infiltration into the nasal mucosa after nasal challenge with OVA. It has been suggested that LFK might modulate the immune response and combat nasal allergy, and our previous study demonstrated that LFK inhibits the peritoneal accumulation of eosinophils.[18] In this study, we used the same dose of LFK, 60 mg/mouse, and first demonstrated the inhibitory effects of LFK on eosinophilic infiltration into the upper airway in a murine model of allergic rhinitis. Other probiotic strains have also been shown to have antiallergic effects. It has been demonstrated that certain probiotic strains attenuate the major characteristics of the asthmatic response in a murine model of allergic airway inflammation[14] and downregulate allergenspecific immune responses.[20] Schiavi[21] showed in a murine model of food allergy that a probiotic mixture significantly reduces symptom scores and histamine release in the feces following allergen challenge and that oral treatment with the probiotic mixture has the capacity to shift a polarized Th2 response to a Th1 type profile. It has been recently shown that perinatal treatment with *Lactobacillus rhamnosus* GG suppresses the development of experimental allergic asthma in adult mice.[22] Furthermore, a few preclinical studies have demonstrated that the use of probiotics is associated with lower symptom scores and medication use,[23] suggesting that probiotics may alleviate allergic rhinitis by reducing symptom severity and medication use and might become an effective strategic option in the prevention and therapy of allergic diseases.

The inhibition of Th2 cytokine release stimulated by allergens and the stimulation of a Th1 cytokine response[21],[24] may be the mechanisms by which probiotics influence allergy. It was reported that some probiotics have the ability to prevent and alleviate allergic disease by inducing a Th1 bias.[25],[26] However, in our study, the oral administration of LFK had no effect on systemic IL-4, IFN-γ, and OVA-specific IgE levels, as evidenced by the levels in both the sera and splenocyte culture supernatants. In addition, IL-4 levels in the saline control mice and IFN-γ levels in the OVA-immunized mice were below the detectable limit. The OVA-specific IgE titer in splenocyte culture supernatants was not detected in all groups probably due to the shorter culture period. Therefore, the ratio of IFN-γ to IL-4, an indication of Th1/Th2 balance, could not be analyzed. As reported by other studies, the regulation of the balance between Th1 and Th2 is important in the control of IgE production[26],[27], suggesting that maybe some other mechanisms might play a role in the anti-allergic effects of LFK.

There is evidence from both experimental and preclinical studies that probiotics may sufficiently stimulate the common mucosal immune system and provide increased protection against allergic airway inflammation[12],[13],[22],[28], food allergy[21],[24], and atopic dermatitis[29],[30]. In particular, there is growing evidence from a variety of model systems that indicates that the ability to induce Tregs and the subsequent attenuation of both Th1 and Th2 responses may be critical in the anti-inflammatory action of many probiotic strains[7],[31],[32]. For instance, oral administration of probiotics (e.g., L. rhamnosus GG, L. reuteri and Bifidobacterium lactis) can increase the proportion of CD4+CD25+ Foxp3+ Tregs and also lead to an enhancement of the regulatory functions of CD4+CD25+ Tregs in the spleens of non-sensitized adult mice, which can lead to the subsequent attenuation of the allergic airway response[32]-[34]. Additionally, Ohno et al.[35] showed that the suppressive effect of B. bifidum G9-1 on OVA-specific IgE production in OVA-immunized mice is due to the reduction of IgE production via an IFN-γ-independent mechanism mediated by Tregs. Some probiotics have the ability to induce Tregs that can attenuate both Th1 and Th2 responses, and Tregs potently suppress IgE production and directly or indirectly suppress the activity of the effector cells, including eosinophils, basophils, and mast cells, of allergic inflammation[31]. Interestingly, the present study showed that the oral administration of LFK for 6 weeks significantly increased the percentage of CD4+CD25+ Tregs in murine spleens, suggesting that CD4+CD25+ Tregs may play a role in the immunomodulatory effects of LFK.

Certain probiotics may induce Tregs in the gut-associated lymphoid tissue that can spread to the airways in response to immune challenge and inflammation[36]. It had been proven that Tregs can migrate to and remain in inflamed tissue and that they were principally involved in the resolution of established inflammation in an animal model of allergic asthma[7],[36],[37]. Transferred CD4+CD25+ Tregs from L. reuteri-fed non-sensitized mice attenuated the allergic airway response in OVA-sensitized mice[34]. Another regulatory mechanism mediated by probiotics in an experimental model involves regulation by Th17, a subset of Th cells[38]. Th17 cells are characterized by their IL-17A, IL-17F, IL-6, tumor necrosis factor-α, and IL-22 expression, and these cytokines coordinate local tissue inflammation through the upregulation of the immunoregulatory cytokine IL-10 and chemokines and the downregulation of proinflammatory cytokines[31],[39]. Presently, there is limited information concerning the effects of probiotics on Tregs in our study, and the local immunoregulatory effects of Tregs in the upper airway are not clear. The precise mechanisms involved will be the subject of further research.

In conclusion, our results indicate that oral administration of LFK may alleviate nasal symptoms and reduce nasal eosinophila in experimental allergic rhinitis. The immunomodulatory effects of LFK may be in part mediated by CD4+CD25+ Tregs, but this mechanism requires further clarification.
